# Scrutinizing the double superconducting gaps and strong coupling pairing in (Li_1−*x*_Fe_*x*_)OHFeSe

**DOI:** 10.1038/ncomms10565

**Published:** 2016-01-29

**Authors:** Zengyi Du, Xiong Yang, Hai Lin, Delong Fang, Guan Du, Jie Xing, Huan Yang, Xiyu Zhu, Hai-Hu Wen

**Affiliations:** 1 Center for Superconducting Physics and Materials, National Laboratory of Solid State Microstructures and Department of Physics, Collaborative Innovation Center for Advanced Microstructures, Nanjing University, Nanjing 210093, China

## Abstract

In the field of iron-based superconductors, one of the frontier studies is about the pairing mechanism. The recently discovered (Li_1−*x*_Fe_*x*_)OHFeSe superconductor with the transition temperature of about 40 K provides a good platform to check the origin of double superconducting gaps and high transition temperature in the monolayer FeSe thin film. Here we report a scanning tunnelling spectroscopy study on the (Li_1−*x*_Fe_*x*_)OHFeSe single crystals. The tunnelling spectrum mimics that of the monolayer FeSe thin film and shows double gaps at about 14.3 and 8.6 meV. Further analysis based on the quasiparticle interference allows us to rule out the *d*-wave gap, and for the first time assign the larger (smaller) gap to the outer (inner) Fermi pockets (after folding) associating with the *d*_*xy*_ (*d*_*xz*_*/d*_*yz*_) orbitals, respectively. The gap ratio amounts to 8.7, which demonstrates the strong coupling mechanism in the present superconducting system.

## Abstract (web summary)

Whilst superconductivity is understood to emerge when electronic pairing allows for conduction without resistance, the mechanism at work in Fe-based superconductors is unclear. Here, the authors address the details of Fermi surfaces and associated gaps as well as the pairing mechanism of (Li_1−*x*_Fe_*x*_)OHFeSe via scanning tunnelling methods.

## Graphical abstract

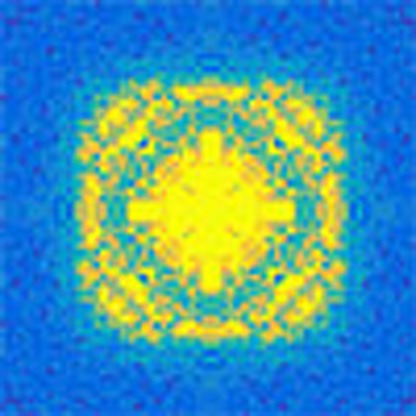

The pairing mechanism and the superconducting gap structure have remained as the core issues in the study on iron-based superconductors. Depending on the atomic structures, the Fermi surface topology changes significantly in different systems, varying from having hole pocket(s) only, to both hole and electron pockets, and to only electron pockets in some FeSe-based systems^1^,^2^,^3^,^4^,^5^,^6^,^7^. In many earlier discovered FeAs- or FeSe-based systems^8^,^9^,^10^ with both electron and hole pockets, a natural pairing picture was proposed^11^,^12^, which concerns the pairing through the pair-scattering process between the electron and hole pockets leading to an extended *s*-wave gap with the pairing manner denoted by *s*^±^. While in the system with absence of the hole pockets, this weak coupling-based picture is facing a great challenge. The question arising immediately is that whether we have a sign change of the gap function as expected from the repulsive interaction-induced pairing^13^, for example, the nodeless *d*-wave with opposite signs between two neighbouring electron pockets. Another puzzling point is that in the scanning tunnelling microscopy/spectroscopy (STM/STS) measurements on the FeSe monolayer thin film^1^ on a SrTiO_3_ substrate, double gaps have been observed on the single tunnelling spectrum with the larger gap ranging from 14 to 22 meV, while in all the angle-resolved photoemission spectroscopy (ARPES) measurements^3^,^4^ there is only one set of electron Fermi pockets near the 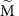 point. It is curious to know how to associate the double gaps with only one set of Fermi pockets. In addition, the larger gap discovered either in STM/STS^1^ or ARPES^3^,^4^ measurements is very big in a sense of weak coupling-based picture. Therefore it was proposed that some interfacial strong phonon mode may join in the electron-boson coupling leading to the high gap value^2^. However, this scenario needs to be verified in systems without the particular substrate and related phonons. It is thus highly desired to do experiments in the FeSe-based system with the similar electronic structures. The recently discovered (Li_1−*x*_Fe_*x*_)OHFeSe phase^5^,^6^,^7^ with a superconducting transition temperature *T*_c_≈40 K shows also only electron pockets around 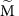 points in a folded Brillouin zone (BZ)^14^,^15^, which is very similar to the case of monolayer FeSe thin film. This provides us a very good platform to check the fundamental problems, such as the origin of the double gaps and whether the weak coupling-based picture holds still for the superconductivity mechanism.

In this paper, we report the STM/STS measurements on the newly discovered superconductor (Li_1−*x*_Fe_*x*_)OHFeSe. It is found that the tunnelling spectrum shows a double gap structure and looks very similar to that observed on the FeSe monolayer thin film. The analysis based on the quasiparticle interference (QPI) technique allows us to carefully scrutinize the superconducting gaps on the associated Fermi surfaces near 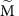 points in (Li_1−*x*_Fe_*x*_)OHFeSe. The larger gap (Δ_1_) derived here is about 14.3 meV, which yields a huge gap ratio of 2Δ_1_/*k*_B_*T*_c_=8.7. This demonstrates the strong coupling mechanism for superconductivity in the present system. Our work shed new light in understanding the puzzling issues in the high-temperature superconducting FeSe monolayer thin films, and will stimulate new efforts in solving the superconductivity mechanism in iron-based superconductors.

## Results

### Characterization of superconducting properties

**Figure 1:**
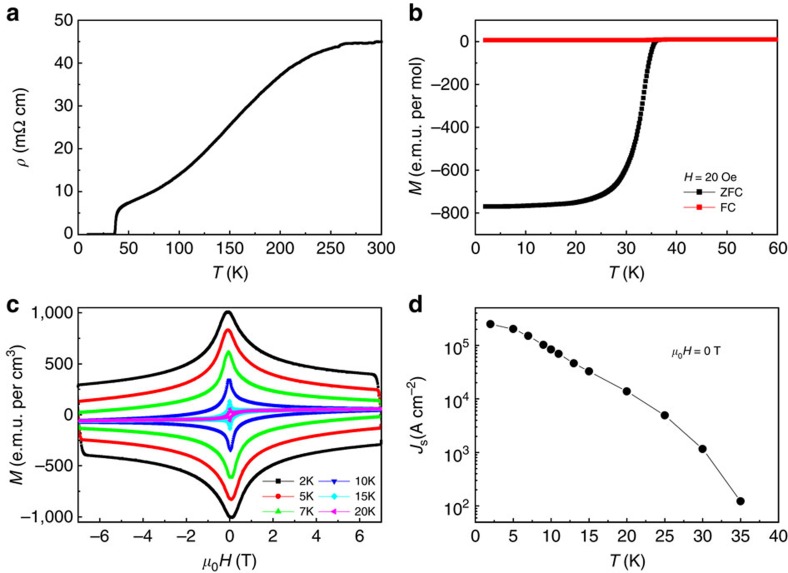
**Superconducting transition and magnetization of the (Li**_**1−** ***x***_ **Fe**_***x***_ **)OHFeSe crystal.** (**a**) Temperature dependence of resistivity of the sample. (**b**) Temperature dependence of DC magnetization measured with the zero-field-cooled (ZFC) and field-cooled (FC) modes at a field of 20 Oe. (**c**) The magnetization-hysteresis-loops measured at temperatures from 2 to 20 K. (**d**) Temperature dependence of the calculated critical current density *J*_s_ based on the Bean critical state model at zero magnetic field.

Figure 1a shows the temperature-dependent resistivity of the (Li_1−*x*_Fe_*x*_)OHFeSe single crystal. One can see that the resistive transition is rather sharp near the foot. The rounded shape near the onset transition is induced by the fluctuating superconductivity since the system is highly two-dimensional (2D). In Fig. 1b, we present the temperature dependence of the DC magnetization of the sample. It is measured at an external magnetic field of 20 Oe with the zero-field-cooled (ZFC) and FC modes. The sample shows perfect Meissner shielding effect with the superconducting transition temperature around 38 K, which is comparable to previous reports^5^,^6^,^7^. The magnetization-hysteresis-loops shown in Fig. 1c exhibit a symmetric feature, which suggests the bulk pinning of vortices and thus the bulk superconductivity of the material. The calculated critical current density at 2 K and 0 T from the Bean critical state model^16^ shown in Fig. 1d is 1–2 orders of magnitude larger than that in K_*x*_Fe_2−*y*_Se_2_ and is comparable to that in the optimally doped Ba(Fe_1−*x*_Co_*x*_)_2_As_2_ (ref. 17). This indicates that the sample is not phase separated as what occurs in K_*x*_Fe_2−*y*_Se_2_. This allows us to have reliable STM/STS measurements.

### Topographic STM image and STS spectra

**Figure 2:**
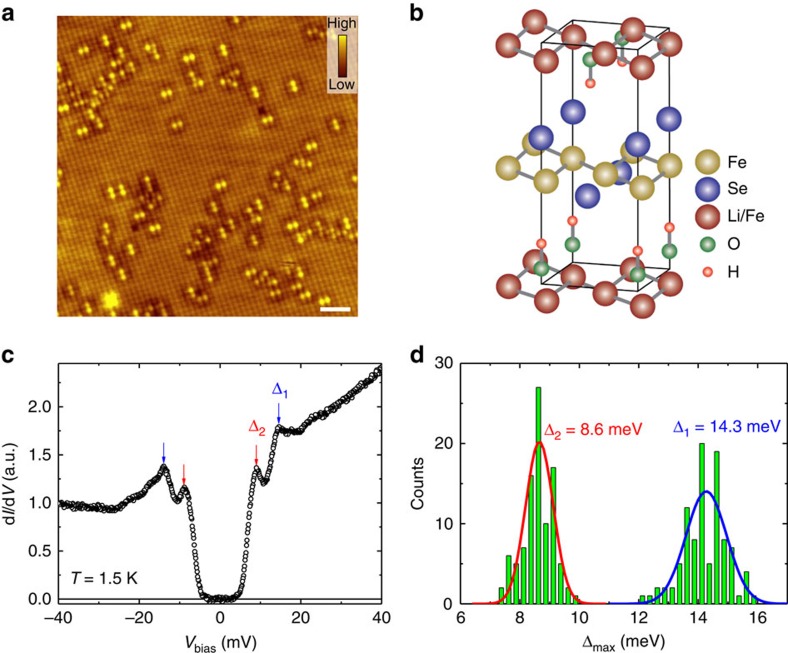
**Topographic STM image and two-gap feature of (Li**_**1−** ***x***_ **Fe**_***x***_ **)OHFeSe.** (**a**) The atomically resolved STM image with a bias voltage of *V*_bias_=180 mV and tunnelling current of *I*_t_=102 pA. The defects with the dumbbell shape are observed on the top surface. Scale bar, 2 nm. (**b**) The schematic atomic structure of (Li_1−*x*_Fe_*x*_)OHFeSe. (**c**) A typical STS spectrum measured at 1.5 K away from the defects. One can clearly see the two-gap feature. Similar STS with double gap structure have been repeated in different areas and different samples. (**d**) Histogram of the superconducting gap values with the fitting results by Gaussian functions (solid lines). Two major gaps can be found.

STM/STS measurements can provide essential information on the electronic structure and the gap symmetry of the novel superconductors^18^,^19^,^20^. Figure 2a shows a topographic image of the terminated surface of the cleaved (Li_1−*x*_Fe_*x*_)OHFeSe single crystal, and the atomically resolved square lattice is clearly seen. The lattice constants at the perpendicular directions are 3.61 and 3.63 Å, respectively, which are comparable to the lattice constant *a* of 3.79 Å from previous reports^5^,^7^. The schematic structure of (Li_1−*x*_Fe_*x*_)OHFeSe is shown in Fig. 2b. Since the cleaved surface is very stable and the lattice constant is very close to the expected value of Se–Se bond, together with the perfect STS measured (see below), we can reasonably assume that the terminated top layer is the Se-atom layer. Some bright spots with a dumbbell shape on the topography are observed and very similar to those from Cu or Co impurities in our previous works^21^,^22^. From the structure of the system, it is found that the impurity atom in the centre of a dumbbell spot locates just at the position of an Fe atom in the beneath layer, and it may be induced by the partial substitution of Fe atoms with Li, or some vacancies at the Fe sites in the Fe layer. This can get support from previous results on LiFeAs in which similar defects on Fe sites were proposed^23^,^24^,^25^. The calculated density of the impurities varies from 0.5 to 1.5% of Fe sites on the FeSe layer, which is much lower than the ratio of about 20% of substitution of Li by Fe atoms in the Li(Fe) layers^5^,^6^,^7^.

Figure 2c shows a typical tunnelling spectrum measured at 1.5 K. Clearly there are two pairs of coherence peaks on the spectrum, indicating existence of two superconducting gaps. The larger gap is marked by Δ_1_ on its peak position of about **±**14.3 meV while the smaller one is marked by Δ_2_ at about **±**8.6 meV. The spectrum with two-gap feature is reminiscent of that reported on the monolayer FeSe thin film on SrTiO_3_ substrate^1^. The recent high-resolution ARPES measurements show only electron pockets as well as one superconducting gap in (Li_1−*x*_Fe_*x*_)OHFeSe^14^,^15^. It should be noted that the two-gap feature in the monolayer FeSe thin film was observed only in the STS measurements^1^, not in the ARPES. This may be induced by the limited resolution of ARPES, or due to some selection rules in the emission process of photo-electrons. More interestingly, the gaps found by the two ARPES measurements from two different groups on the similar samples are corresponding to the larger^14^ and smaller gap^15^ maxima of our STS measurements, respectively. The two-gap feature is a common one in our (Li_1−*x*_Fe_*x*_)OHFeSe samples, which can get support from the statistical result (Fig. 2d) of the peak positions on 98 spectra collected randomly in the range of 41 nm × 41 nm. By fitting to the Gaussian functions, we obtained the two-gap values of Δ_1_=14.3±1.3 and Δ_2_=8.6±0.9 meV, and the corresponding gap ratios of 2Δ_1_/*k*_B_*T*_c_=8.7±0.8 and 2Δ_2_/*k*_B_*T*_c_=5.2±0.5. These values are much larger than the one 2Δ/*k*_B_*T*_c_∼3.53 predicted by the Bardeen–Cooper–Schrieffer (BCS) theory in the weak coupling regime. The huge value 2Δ_1_/*k*_B_*T*_c_=8.7 indicates strong coupling mechanism in the system. Worthy of noting is that similar ratio was observed in the cuprate superconductors^26^,^27^.

### Temperature-dependent STS spectra and Dynes model fitting

**Figure 3:**
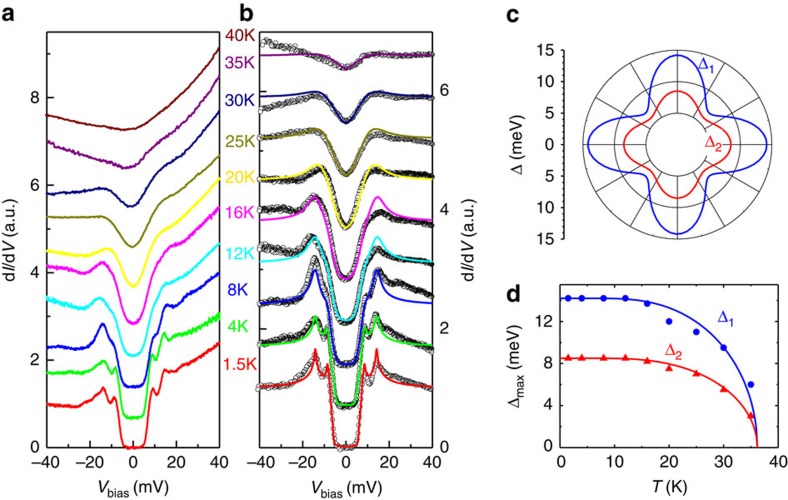
Temperature-dependent tunnelling spectra and theoretical fits. (**a**) The evolution of the STS spectra measured at temperatures from 1.5 to 40 K. (**b**) Fitting results to the STS spectra normalized by the one measured at 40 K in the normal state. The dark hollow circles represent the experimental data, and the coloured solid lines are the theoretical fits to the data with two anisotropic *s*-wave gaps by the Dynes model. (**c**) The anisotropic-gap functions used in the fitting to the curve measured at 1.5 K. (**d**) Temperature dependence of the two gaps extracted from the Dynes model fitting, and the solid lines denote the theoretical calculations of the superconducting gap from the BCS model.

**Figure 4:**
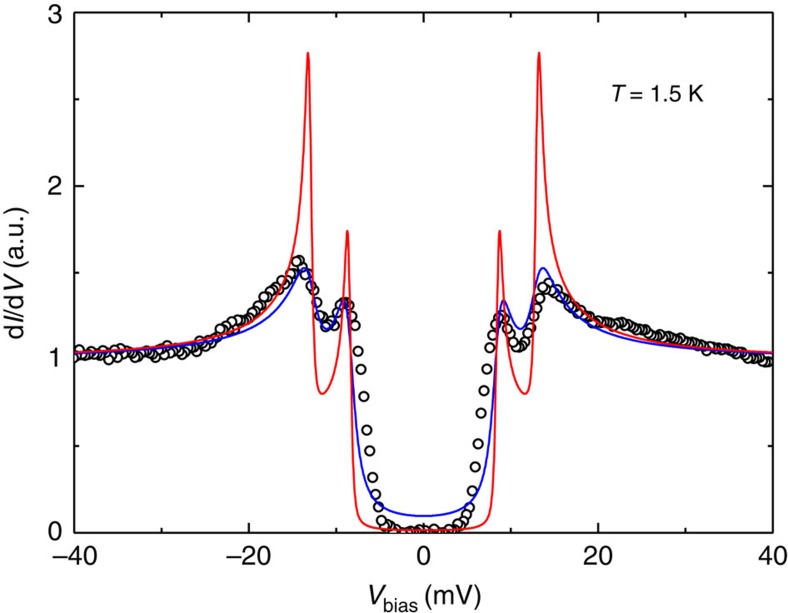
**Theoretical fitting results by two isotropic** ***s*** **-wave gaps.** The fitting results shown as solid lines with different fitting parameters fail to catch up the main features of the experimental data. For the fittings, Δ_1_=13 meV and Δ_2_=8.5 meV, *Γ*_1_=*Γ*_2_=0.15 meV were used for the clean case (red line), while the same gaps and *Γ*_1_=1.2 and *Γ*_2_=0.85 meV for the case of finite scattering rates (blue one). From these fittings, one can see that the tunnelling spectrum cannot be fitted with two isotropic gaps.

In Fig. 3a, we show the temperature evolution of the tunnelling spectra measured at the same position. The coherence peaks associating with both gaps are suppressed and mix together with increasing temperature, and finally vanish above *T*_c_. To see the possible influence of the electronic bands on the tunnelling spectrum, we show one STS measured in wide energy ranges in Supplementary Fig. 1. The spectra shown in Fig. 3b are normalized (divided by the one measured at 40 K). The two well-resolved coherence peaks together with the fully gapped density of states (DOS) near zero-bias allow us to make reliable fitting using the Dynes model^28^. From the normalized spectrum at 1.5 K, one can see that the bottom of the STS is very flat and has a zero value, however, the coherence peaks are not very sharp. Together with the latter effect is the finite slope of the rising edges of the STS near the bottom. To know whether these contradicting phenomena are induced by the finite scattering rates, we firstly try to use two isotropic *s*-wave gaps and different pairs of scatterings rates *Γ*_1(2)_ to fit the experimental data. Because of the two-gap feature, in the model to fit the normalized spectra, the differential conductivity is constructed as: *G*=*x*d*I*_1_/d*V*+(1−*x*)d*I*_2_/d*V*, where *I*_1(2)_(*V*) is the tunnelling current contributed by the larger(smaller) gap, and *x* is the related spectral weight. The detailed fitting process and parameters are given in Supplementary Note 1. The fitting results with two isotropic *s*-wave gaps under zero and finite scattering rates are presented in Fig. 4. It is clear that, even with the finite scattering rates, the fitting with isotropic gaps fails to catch up the main features of the experimental data. We then use the two anisotropic *s*-wave gaps 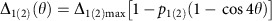 to fit the experimental data and show the results in Fig. 3b. One can see from Fig. 3b that the theoretical model fits the experimental data very well. The fitting process is detailed in Supplementary Note 1 and the fitting parameters are listed in Supplementary Table 1. The fourfold symmetric gap functions used to fit the data at 1.5 K are presented in Fig. 3c, and the anisotropy *p* is fixed in all the fittings, that is, 25% for Δ_1_(*θ*) and 15% for Δ_2_(*θ*), while the weight of the larger gap Δ_1_(*θ*) is fixed with the value of 65% at different temperatures. The resultant gap maxima versus temperature are shown in Fig. 3d, and the solid lines are the results of theoretical calculation within the BCS model by fixing Δ_max_(0) and *T*_c_ derived from our experiment. One can see that the temperature-dependent superconducting gaps can be well-described by the BCS theory. Our results suggest that this material has two nodeless superconducting gaps, each with a significant anisotropy. The two-gap feature is difficult to be understood if only one set of electron Fermi pockets around the 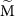 point exists as observed in the ARPES measurements.

### QPI measurements

**Figure 5:**
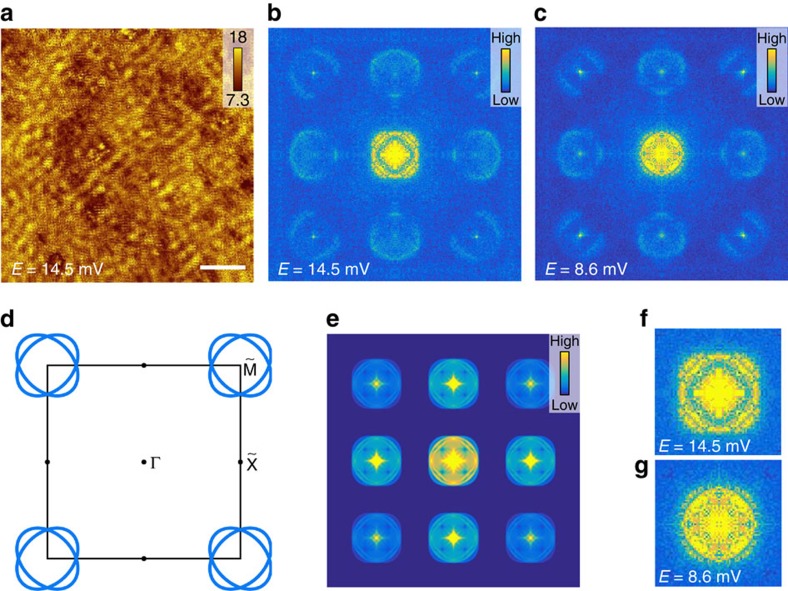
Quasiparticle interference patterns and theoretical simulation. (**a**) QPI image *g*(**r**, *E*=14.5 mV) in real space measured at 1.7 K near the energy of the coherence peak position of the larger gap. Scale bar, 5 nm. (**b**,**c**) FT-QPI intensity *ρ*(**q**, *E*) obtained by taking Fourier transformations on the corresponding real-space image measured with energies near the coherence peak position of the two gaps, namely 14.5 and 8.6 mV. The images are fourfold symmetrized to enhance the signal. (**d**) Schematic plot of the elliptic-shaped electron pockets in folded Brillouin zone. (**e**) The theoretical simulation of the QPI scattering intensity by applying autocorrelation to **d**. (**f**,**g**) Zoom-in images of the central parts of **b** and **c**, which contain the information of the small-*q* intra-pocket scattering. The image at 14.5 mV is very similar to the structure of the central pattern in (**e**). However the central pattern at 8.6 mV has a very different shape, and the outer ring seems to be less pronounced.

On the surface of the crystal, we can see some dumbbell-shape impurities. The spectrum measured on the impurity site has no well-defined superconducting coherence peaks as shown in Supplementary Note 2. Instead, we can see the in-gap states with two asymmetric peaks which make the bottom near zero-bias ‘V'-shaped (Supplementary Fig. 2). The impurities which act as the scattering centres will produce the standing waves, and such QPI imaging can provide fruitful information on the scattering between the contours of Fermi surfaces with the scattering vector **q** at constant energy *E* in **k**-space^20^. We perform the QPI measurements on two areas with different scanning range as their topographies shown in Supplementary Fig. 3a,b. The details of the measurements of QPI and data treatments are given in Supplementary Note 3. The standing waves can be clearly seen in Fig. 5a measured around the larger energy gap Δ_1_. When we do the Fourier transformation (FT) to the QPI image, the resultant *ρ*(**q**, *E*) image, or called as the FT-QPI image, shown in Fig. 5b,c helps us to investigate the Fermi surfaces and the superconducting gaps of the material. The resultant FT-QPI pattern looks quite similar to the data on the monolayer FeSe thin film on SrTiO_3_ (001) as recently revealed by the STM measurement^29^. Quite interestingly, the contours near the corner of the FT-QPI pattern corresponding to the large-**q** scattering have clear anisotropic feature, which was also observed on the monolayer FeSe thin film on SrTiO_3_ (001) and was attributed to the orbital features^29^. The ARPES^14^,^15^ measurements show only one set of electron pockets near 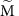 points. However, if taking a closer look at the central part of the FT-QPI images as shown in Fig. 5f,g, we can find that it is unlikely. There are actually two circles, which means that a single set of isotropic ring-shaped Fermi surfaces would not be possible to produce such patterns. Therefore we use elliptic electron Fermi surfaces to simulate the QPI image. Because there are two Fe sites and two distinct Se sites in one unit cell, the two slightly elliptic Fermi pockets will naturally fold into the smaller BZ, as sketched in Fig. 5d. In the picture of folded Fermi surfaces, we can consider the Fermi pockets as inner and outer rings. Due to the possible spin-orbital coupling, the two Fermi pockets may further hybridize into two sets of Fermi pockets with inner and outer contours^30^. We thus do the QPI simulation of this type of Fermi pockets and the resultant pattern (Fig. 5e) is comparable with our experimental data. As shown in Fig. 5b,c, when the bias voltage was increased from Δ_2max_=8.6 to Δ_1max_=14.5 meV, the peripheral patterns from the inter-pocket scattering have negligible difference while the pattern in the centre corresponding to the intra-pocket scattering changes a lot. The magnitude of the outer circle in the central pattern (Fig. 5f,g) seems to be enhanced obviously when the energy increases from the small energy gap to the larger one. The intensity of the FT-QPI contour reflects the joint DOS (JDOS) between two **k**-points on the Fermi surfaces. The emergence and enhancement of intensity in the contour in **q**-space of the outer circle at a higher energy suggests that the superconducting gap on this Fermi surface is larger than that of the inner one^31^,^32^,^33^.

### Intraband QPI

**Figure 6:**
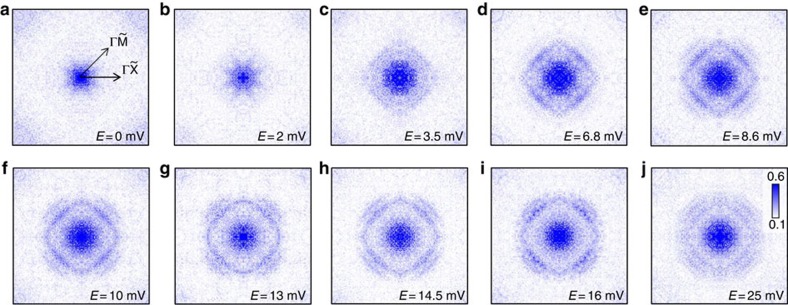
**The evolution of FT-QPI intensity** ***ρ*** **(q,** ***E*** **) at different energies.** (**a**–**j**) The FT-QPI *ρ*(**q**, *E*) images are derived from Fourier transformation on the QPI images with the real-space scale of 58 nm × 58 nm. A 2D-gaussian-function background was subtracted from the raw FT-QPI image and the fourfold symmetrization was carried out to enhance the signal. The intensity on the inner ring in **q**-space shows up at above 3.5 mV, the ring becomes clear at the energy of the smaller gap, namely 8.6 mV, while the segments corresponding to the outer ring appear gradually with a higher energy above 6.8 mV, and are clear when measuring with the voltage at the larger energy gap 14.5 mV.

**Figure 7:**
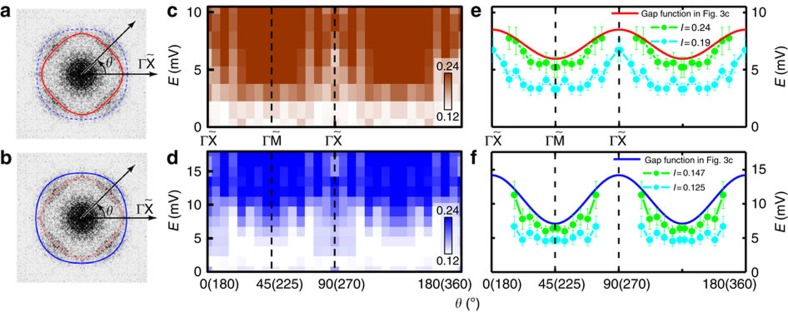
2D colour plots of FT-QPI intensity *ρ*(*θ*, *E*) along the inner and outer contours of the central pattern in q-space. (**a**,**b**) The FT-QPI pattern taken at 14.5 mV. The solid-line contours in **a** and **b** were expressed approximatively by *R*_1_−*r*_1_cos4*θ* and *R*_2_+*r*_2_cos4*θ* for outer and inner rings, respectively. (**c**,**d**) The FT-QPI intensity along the inner (**a**) and outer (**b**) rings at different angles taken from the fourfold symmetrized FT-QPI patterns in Fig. 6 with various energy values. The initial polar angle starts from the 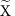 direction. The gap minimum locates in the 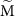 direction for both contours. (**e**,**f**) The angle-dependent energy thresholds taken from the vertical line-cuts to the colour plots in **c**,**d** by using the intensity *I*=0.19, 0.24 (0.125, 0.147) for the inner (outer) rings in **a**,**b**, respectively. The gap minimum locates in the 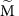 direction for both contours. The solid lines in **e**,**f** plots are the gap functions derived from the Dynes model fitting to the spectrum taken at 1.5 K. The error bars were determined from the deviations between the line-cut experimental data and the polynomial fitting curve for each angle.

We then pay attention to the central pattern by measuring the QPI mapping in a larger real-space scale to get the detailed features of the smaller **q**-space view, and the evolution of the *ρ*(**q**, *E*) images is shown in Fig. 6. The raw data of the related QPI images are given in Supplementary Fig. 4. One can see that there is almost nothing at the locations of the two interference rings at the energy of 0 and 2 mV. This may rule out the existence of any gap nodes on the Fermi surface, since the FT-QPI images would show an intensity at the **q**-vector connecting two nodal points if they would exist. This is also consistent with the full gap structure as seen from the STS spectrum. At the energy of 6.8 mV, a ring with enhanced intensity on the FT-QPI images can be observed, with the strongest intensity along the 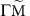 direction. At the energy of the small gap, namely 8.6 mV, the FT-QPI pattern looks similar to that at 6.8 mV. However, when the energy is further increased to the larger gap, that is, 14.5 mV, besides the inner ring, a new set of segments appears at larger **q** vectors, which gradually forms an outer circle. When we take the *ρ*(**q**, *E*) intensity along the contours of the inner and outer rings as shown in Fig. 7a,b, an angle-dependent *ρ*(*θ*, *E*) at various energies can be obtained and presented in Fig. 7c,d as the 2D colour plots. The 2D colour plots of *ρ*(*θ*, *E*) along the inner and the outer rings both show the fourfold symmetry. The line-cut of QPI intensity versus **q** along the radial direction at several angles is shown in Supplementary Fig. 5. The low or null intensity of *ρ*(*θ*, *E*) near the 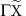 direction along each ring may be induced by the band-crossing effect, or suggest that the Fermi surface in this part is still gapped, so both minima of the gaps appear in the 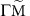 directions. To illustrate it more clearly, we take the vertical line-cuts on the colour plot and show in Fig. 7e,f the angle-dependent energy thresholds at two certain FT-QPI intensities along the inner and outer rings. Considering the two anisotropic *s*-wave gap functions derived in the Dynes model fitting to the measured STS spectrum, we put the angle-dependent gap functions, as shown by the solid lines in Fig. 7e,f, onto the vertical line-cut data (shown by the symbols) at two certain *ρ*(*θ*, *E*) intensities (*I*=0.19, 0.24 and *I*=0.125, 0.147 for the inner and outer rings, respectively). If taking the values of *I*=0.24 and 0.147 as the rough criterion for the gaps on the inner and outer pockets, one can see that the gap functions follow those derived from the Dynes model fitting pretty well. Both gap minima on the two circles appear in the 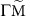 directions, which is consistent with the direction of gap minima of electron pockets in FeSe_0.45_Te_0.55_ (ref. 34). In the narrow region along the 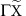 direction, since the intensity is very low and the uncertainty is quite large, we are not able to derive the reliable gap values. Through above analysis and discussions, we can assign the larger (smaller) gap Δ_1_ (Δ_2_) to the outer (inner) Fermi surfaces, which form two circles in the FT-QPI pattern in the **q**-space.

## Discussion

Concerning the two-gap functions with different maximum magnitudes derived from the fitting to both STS spectra and the emergence of two sets of rings in the FT-QPI images, we would like to put forward the picture concerning the band folding (perhaps also hybridization) of the two electron pockets in the folded BZ, as shown in Fig. 5d. In iron-based superconductors, the electron pockets are usually elliptic-shaped and then fold (perhaps also by hybridization) in the folded BZ with two Fe atoms in one unit cell. On the single elliptic Fermi surface, the segments in the pole region are contributed by the *d*_*xy*_ orbitals, while the waist areas are derived from the *d*_*xz*_/*d*_*yz*_ orbitals^35^. Due to the folding effect, we have two sets of Fermi pockets which correspond well to the inner (*d*_*xz*_*/d*_*yz*_) and outer (*d*_*xy*_) pockets. The crossing area consists of electrons with mixed orbitals of *d*_*xy*_ and *d*_*xz*_/*d*_*yz*_. This may be the reason why JDOS is very weak between the overlapped Fermi surfaces and the outer ring seems not to close completely in the 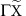 directions. The anisotropic FT-QPI intensity has been self-consistently explained as the cause of the gap anisotropy in present study. While it may also be influenced by (or partly related to) the variations in the orbital character of the bands around a given energy contour^25^,^29^,^31^, and the unclosed part along 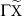 directions mentioned above may give such suggestion. We cannot rule out the effect of the anisotropic orbital character to the anisotropic FT-QPI patterns. Another possible reason for the anisotropic FT-QPI intensity is the anisotropic self-energy, which can affect the FT-QPI pattern, especially when the measuring energy is above the superconducting gap^36^. In the low energy FT-QPI, such effect may also appear together with the anisotropic superconducting gap^36^, although this could be a much weaker effect. Since the anisotropic-gap function is strongly tied with the anisotropic orbital character and self-energy effect, it will be important to figure out which factor is the dominant role for inducing the anisotropic FT-QPI intensity, especially in the energy region above the larger gap. We must emphasize that all these possible alternatives do not change the major results in this study, that is, the identifying of the double electron pockets near 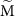 point and the associated double gaps. If the nodeless *d*-wave is applicable in the system, sign reversal of superconducting gaps should exist between two neighbouring elliptic Fermi pockets. In this case, in the band folding (perhaps also hybridized) region, the gap should be zero, which is not consistent with our results. In this picture with electron pocket folding, our data indicate a smaller gap in the inner pocket with the *d*_*xz*_*/d*_*yz*_ characteristic, while the larger gap accommodates in the outer pocket with the *d*_*xy*_ characteristic. To our knowledge, this is the first time to resolve the two sets of Fermi pockets and associated gaps near the 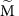 point. In the situation of *s*-wave gap on the hybridized electron pockets, the theoretically predicted gap maximum locates in the 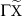 directions and gap minimum locates in the 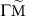 directions^13^, which is consistent with our results. Although we cannot recognize that the gap maxima locate in the 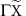 directions just from the intensity plot of FT-QPI in Fig. 7c–f, however, the tunnelling spectrum shows fully gaped feature near zero-bias together with a fourfold angle dependence of the gap, the FT-QPI shows also a fourfold feature and the gap minimum locates in the 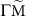 directions. All these strongly suggest that the gap distribution may be very similar to the situation shown in Fig. 7c–f. Further ARPES experiments with refined resolution are strongly desired to confirm the conclusions derived here. In addition, our results here resolve two sets of Fermi pockets and associated gaps, which provides a very good stage for further checking whether there are sign reversal gaps between the inner and outer folded (or hybridized) Fermi pockets. This is actually underway by following the recent theoretical predictions^37^. That should be very crucial for a final pinning down of the repulsive interaction-induced pairing. Our observation of two superconducting gaps and the assignment of the two gaps to the folded (or hybridized) two sets of Fermi pockets in the new superconductor (Li_1−*x*_Fe_*x*_)OHFeSe is very intriguing. Furthermore, the huge gap ratio 2Δ_1_/*k*_B_*T*_c_=8.7 indicates the strong coupling mechanism for superconductivity in the iron-based superconductors.

## Methods

### Sample synthesis and characterization

The (Li_1−*x*_Fe_*x*_)OHFeSe single crystals were synthesized by the hydrothermal ion-exchange method^7^. First, 6 g LiOH (J&K, 99% purity) was dissolved in 15 ml deionized water in a teflon-linked stainless-steel autoclave (volume 50 ml). Then, 0.6 g iron powder (Aladdin Industrial, 99% purity), 0.3 g selenourea (Alfa Aesar, 99% purity) and several pieces of K_0.8_Fe_2−*x*_Se_2_ single crystals were added to the solution. After that, the autoclave was sealed and heated up to 120 °C followed by staying for 40 to 50 h. Finally, the (Li_1−*x*_Fe_*x*_)OHFeSe single crystals can be obtained by leaching. The DC magnetization measurement was carried out with a SQUID-VSM-7T (Quantum Design) with a resolution of 5 × 10^−8^ e.m.u. The resistivity measurement was done on a PPMS-16T (Quantum Design) with the standard four-probe method.

### STM/STS measurements

The STS spectra were measured with an ultra-high vacuum, low-temperature and high-magnetic field scanning tunnelling microscope (USM-1300, Unisoku Co., Ltd.). The samples were cleaved in an ultra-high vacuum with a base pressure about 1 × 10^−10^ torr. During all STM/STS measurements, tungsten tips were used. To lower down the noise of the differential conductance spectra, a lock-in technique with an ac modulation of 0.8 mV at 987.5 Hz was used.

## Additional information

**How to cite this article:** Du, Z. *et al.* Scrutinizing the double superconducting gaps and strong coupling pairing in (Li_1−*x*_Fe_*x*_)OHFeSe. *Nat. Commun.* 7:10565 doi: 10.1038/ncomms10565 (2016).

## Supplementary Material

Supplementary Information Supplementary Figures 1-5, Supplementary Table 1 and Supplementary Notes 1-3
