# Supplementary material for: Scrutinizing the double superconducting gaps and strong coupling pairing in (Li1−xFex)OHFeSe
Source: Nat Commun. 2016 Jan 29;7:10565. doi: 10.1038/ncomms10565 (PMC4740187; doi:10.1038/ncomms10565)
Supplement: Supplementary Information — Supplementary Figures 1-5, Supplementary Table 1 and Supplementary Notes 1-3 [file ncomms10565-s1.pdf]

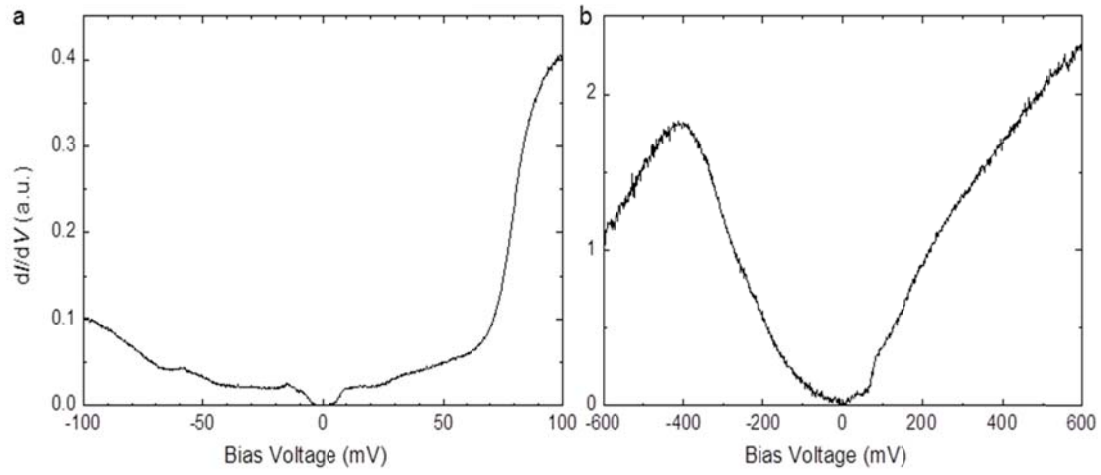

Supplementary Figure 1 | STS spectra measured at 1.5 K in the large bias voltage ranges. A broad peak locates at about -400 mV and a sharp increase is observed above 70 mV, which may be related to the band structure.

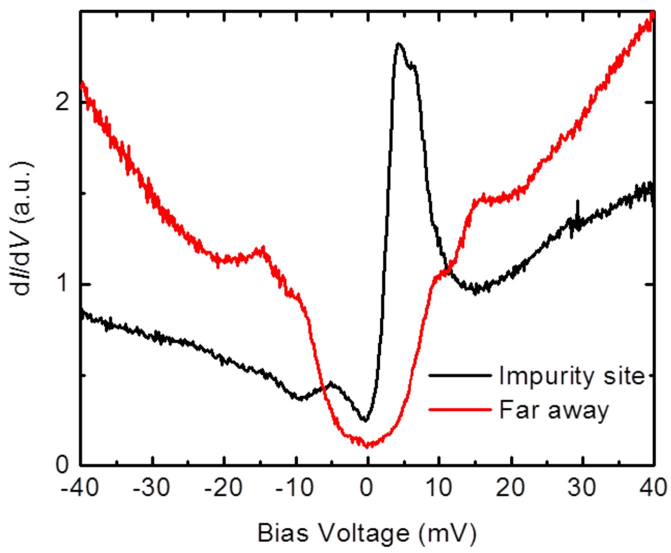

Supplementary Figure 2 | STS spectra measured on the impurity site (black curve) and the clean region (red curve) far away from impurities.

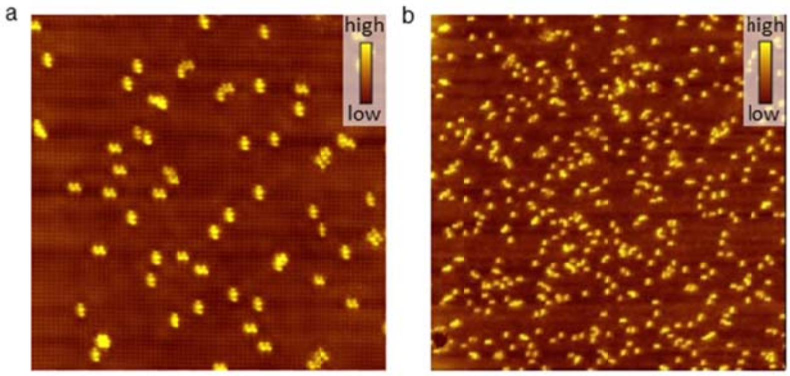

Supplementary Figure 3 | Atomically resolved topographies with different scanning ranges. (**a**), A topography image of 28 nm × 28 nm (measuring conditions V_{bias} = 40 mV, I_t = 100 pA). The QPI image on the same area is shown in Fig. 5. (**b**), A topography image (V_{bias} = 40 mV, I_t = 102 pA) measured in another region with area of 58 nm × 58 nm, and the QPI image on the same area is shown in Fig. 6.

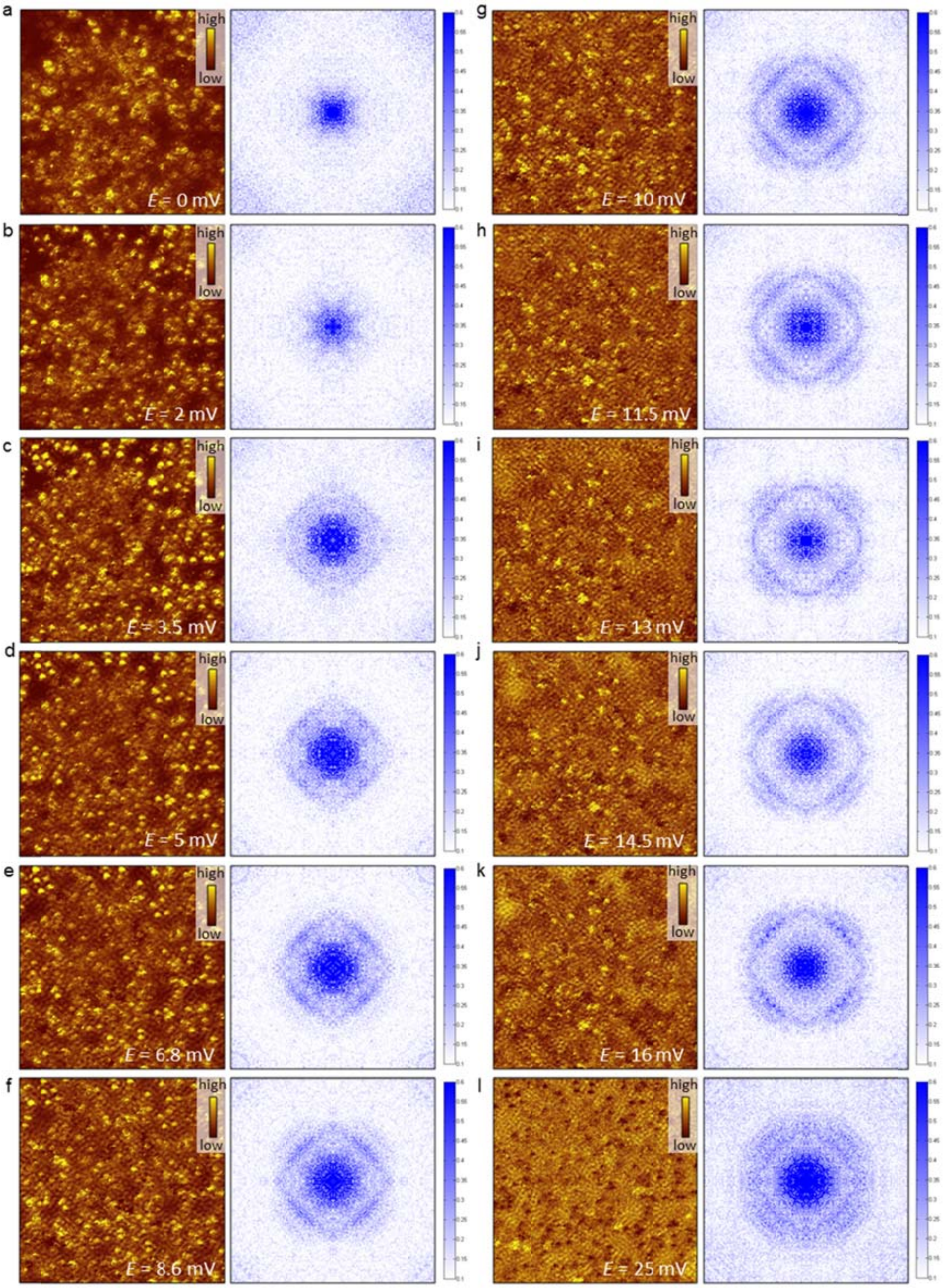

Supplementary Figure 4 | Sequence of the QPI images and their respective FT-QPI images. The QPI images (**a**-**l**) in real space were taken in a 58 nm × 58 nm area.

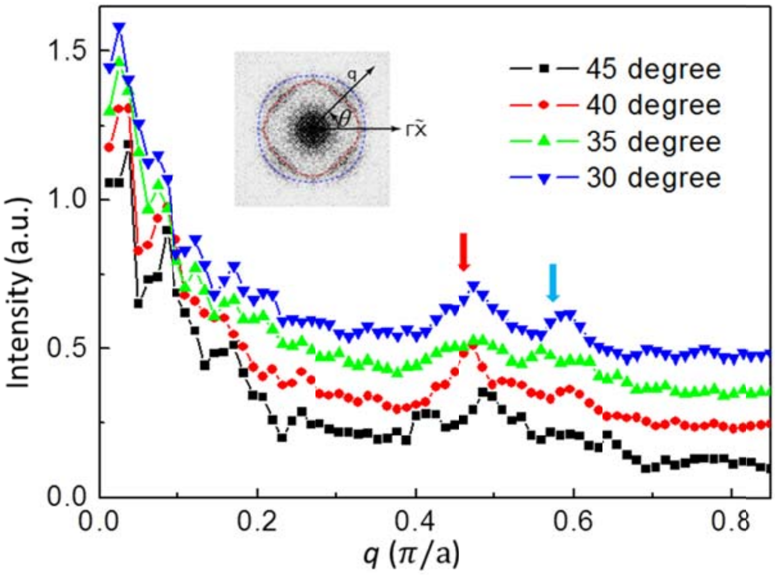

Supplementary Figure 5 | The line-cut of QPI intensity versus q along the radial direction. The **q**-dependent FT-QPI intensity at four angles θ=30, 35, 40 and 45° (with offsets). Two intensity peaks can be observed from both the FT-QPI pattern (inset) and the line-cut near the $\Gamma\widetilde{M}$ direction. The polar angle counts from the $\Gamma\widetilde{X}$ direction.

Supplementary Table 1 | Fitting parameters to the experimental data at different temperatures by using two anisotropic *s*-wave gaps.

T (K)	Δ_{1max} (meV)	Δ_{2max} (meV)	Γ_1 (meV)	Γ_2(meV)
1.5	14.2	8.5	0.35	0.2
4	14.2	8.5	0.35	0.1
8	14.2	8.5	0.05	0.05
12	14.2	8.5	0.2	0.15
16	13.9	8.3	0.2	0.15
20	12	7.7	1	1
25	11.7	7.2	2	2
30	9.5	5.5	2.7	2.3
35	6	3	3	3

Supplementary Note 1: STS measurements and theoretical fitting

The tunnelling spectra exhibit a two-gap feature and zero conductance near the zero-bias. So we firstly use two isotropic *s*-wave gaps with different pairs of scatterings rates $\Gamma_{1(2)}$ to fit the experimental data. In the model to fit the normalized spectra, the differential conductivity is constructed as: $G = x dI_1/dV + (1-x) dI_2/dV$, where $I_{1(2)}(V)$ is the tunnelling current contributed by the larger(smaller) gap, and x is the related spectral weight. $I_{1(2)}(V)$ is given by

$$I_{1(2)}(V) = \int_{-\infty}^{+\infty} d\varepsilon \left[f(\varepsilon) - f(\varepsilon + eV) \right] \cdot \mathrm{Re} \left\{ \frac{\varepsilon + eV + i\Gamma_{1(2)}}{\sqrt{(\varepsilon + eV + i\Gamma_{1(2)})^2 - \Delta_{1(2)}^2}} \right\}. \quad (1)$$

Here, $f(\varepsilon)$ is the Fermi function containing the information of temperature, and $\Gamma_{1(2)}$ is the scattering factor of the larger (smaller) gap, and the result is shown in Fig. 4. One can see that the fitting result fails to catch up the main features of the experimental data with two isotropic gaps, even considering the different scattering rates. So we instead use two anisotropic *s*-wave gaps to fit the data, and in this situation $I_{1(2)}(V)$ reads

$$I_{1(2)}(V) = \frac{1}{2\pi} \int_{-\infty}^{+\infty} d\varepsilon \int_{0}^{2\pi} d\theta \left[f(\varepsilon) - f(\varepsilon + eV) \right] \cdot \mathrm{Re} \left(\frac{\varepsilon + eV + i\Gamma_{1(2)}}{\sqrt{(\varepsilon + eV + i\Gamma_{1(2)})^2 - \Delta_{1(2)}^2(\theta)}} \right). \quad (2)$$

We take the anisotropic nodeless gap function $\Delta_{1(2)}(\theta) = \Delta_{1(2)\max}\left[1 - p_{1(2)}(1 - \cos 4\theta)\right]$ in which p determines the anisotropy of gaps. In all process of fitting, $\Delta_1(\theta) = \Delta_{1\max}(0.25\cos 4\theta + 0.75)$ and $\Delta_2(\theta) = \Delta_{2\max}(0.15\cos 4\theta + 0.85)$, respectively. The fitting parameters for the normalized spectrum shown in Fig. 3b are listed in Supplementary Table 1. The spectral weight of the larger gap is fixed to 65% at different temperatures.

STS spectra measured in the wide energy ranges are shown in Supplementary Figure 1 from which one can get some information on the band structure.

Supplementary Note 2: Impurity-induced in-gap states

A typical STS spectrum in Supplementary Figure 2 shown as the red line was

measured far away from the impurity site. The black curve in Supplementary Figure 2 represents the curve measured at the center of a dumbbell shape defect, which shows strong suppression of the coherence peaks as well as clear in-gap impurity states marked by two asymmetric resonance peaks with the peak positions at around ± 5 mV.

Supplementary Note 3: QPI measurements and data treatments.

We performed the QPI measurements in two areas with different scanning ranges as their topographies shown in Supplementary Figures 3a and 3b. The corresponding QPI images measured at various energies are shown in the main text Fig. 5 and Fig. 6. The large scanning area helps us to investigate the intra-pocket scattering.

To reduce the noise of the scattering patterns from the Fourier transformation, we suppress the intensity in the center by multiplying a 2D factor of $1-A\times g[\mathbf{q}(0,0),\sigma]$ to the raw FT-QPI images. Here $g[\mathbf{q}(0,0),\sigma]$ is a 2D Gaussian function with the center at central point $\mathbf{q}(0,0)$ and the standard deviation of σ. The parameters A and σ were adjusted slightly to make the intensities around a circle far away from the central pattern at different energies the same magnitude. Then we do the fourfold symmetrization to the resultant images. Supplementary Figure 4 shows the evolution of QPI images and the corresponding FT-QPI images at different energy values.

In Supplementary Figure 5, we show the $\mathbf{q}$-dependent FT-QPI intensity at four angles θ=30, 35, 40 and 45° (with offsets). One can see two intensity peaks from the line-cut data near the $\Gamma\widetilde{\mathrm{M}}$ direction.
